# Current status of elevated blood pressure and hypertension among adolescents in Asia: a systematic review

**DOI:** 10.7189/jogh.15.04115

**Published:** 2025-03-28

**Authors:** Binish Islam, Tasiu Ibrahim Ibrahim, Wang Tingting, Mingyang Wu, Qin Jiabi

**Affiliations:** 1 Xiangya School of Public Health, Central South University, Changsha, China; 2 Department of Neurological Surgery, the Second Xiangya Hospital of Central South University, Changsha, China; 3 School of Public Health, Kunming Medical University, Kunming, China

## Abstract

**Background:**

Hypertension among adolescents in Asia is an emerging public health concern that is directly associated with early onset cardiovascular risks. As such, it can also lead to further health issues and challenges for health care in the future. As existing studies have predominantly focussed on adult populations, we sought to provide targeted insights into adolescent hypertension across Asia, elucidating the impact of rapid lifestyle and environmental changes on this younger population. Therefore, in this systematic review, we aimed to evaluate the prevalence and trends of elevated blood pressure (BP) and hypertension among adolescents aged 10–19 years across Asia, address gaps in region-specific data, and determine any demographic risk factors.

**Methods:**

Following PRISMA guidelines, we searched PubMed, EMBASE, Science Direct, Web of Science, Google Scholar, and Scopus for cross-sectional studies on adolescent hypertension/elevated BP in Asia published from January 2019 to June 2024, after which we narratively synthesised their findings.

**Results:**

Of the 2634 retrieved studies, 39 met the inclusion criteria, covering over 200 000 adolescents in Asia. The prevalence of hypertension ranges from 0.7% in urban Bangladesh to 24.5% in urban Malaysia, with urban areas generally showing higher rates than rural areas (*e.g.* India: 8.4% urban *vs*. 5.7% rural). By region, East Asia has the highest overall prevalence (14.25%), followed by West Asia (14.14%), South Asia (13.77%), Southeast Asia (13.16%), and Central Asia (12.37%). Males had higher prevalence rates (for example, 22.3% in Chinese males *vs.* 20% in females).

**Conclusions:**

The increasing prevalence of adolescent hypertension in urban Asia is a significant public health concern. Although extensive research has been conducted in East and South Asia, there is a dearth of studies in Western, Southeast, and Central Asia, emphasising a need for future research. Standardised diagnostic criteria and targeted interventions are crucial for addressing regional disparities and reducing long-term cardiovascular risks.

Hypertension is a critical public health issue and a leading cause of cardiovascular disease, morbidity, and premature mortality worldwide [1]. About 1.13 billion people – one in six globally – are affected by high blood pressure (BP), a figure which is projected to increase to 1.5 billion by 2025 [2]. Despite growing awareness, less than 20% of individuals with hypertension manage to control it [3]. This poor management leads to serious outcomes, contributing to more than 9.4 million deaths every year [4]. While hypertension is typically recognised as an adult health issue, its rising prevalence among adolescents demands separate attention due to unique physiological, behavioural, and environmental factors that shape their health outcomes [5]. These factors make adolescents particularly vulnerable, necessitating focussed attention distinct from studies of adults, as early interventions during this period offer a unique opportunity to alter long-term health trajectories [6,7].

Currently, approximately 8% to 10% of adolescents globally are affected by hypertension, a prevalence that is anticipated to increase concomitantly with rising obesity rates and increasingly sedentary lifestyles.[8]. Hypertension in this age group has been diagnosed by using distinct clinical criteria. According to the American Academy of Pediatrics (AAP), individuals aged 13 years or older are classified as hypertensive if their systolic blood pressure (SBP) reaches or exceeds 130 mm Hg, or if their diastolic blood pressure (DBP) is 80 mm Hg or higher, as confirmed through two separate readings [9]. Early onset of hypertension has serious long-term implications, with research indicating that adolescents with elevated BP are up to four times more likely to develop cardiovascular complications such as coronary artery disease, stroke, and heart failure later in adulthood [10]. Regular screening and lifestyle modifications are crucial for controlling BP and reducing the future burden of cardiovascular disease [11].

In recent decades, the prevalence of hypertension has surged globally due to shifts in diet, declining physical activity, and rapid urbanisation [12]. In Asia, nearly 30% of adults are affected by hypertension, and countries such as Malaysia, Thailand, and Indonesia experience significant burden [13]. Emerging data show that 10–15% of adolescents in urban areas across Southeast Asia already exhibit elevated BP, reflecting patterns observed among adults [14]. Lifestyle changes, such as reduced physical activity and poor dietary habits, are key drivers of this growing concern among youth, highlighting the urgent need for early intervention [15]. Furthermore, gender and socioeconomic status also impact on the prevalence of hypertension [16], with disparities reported in urban *vs.* rural settings and differing patterns among boys and girls during puberty [17]. Despite the growing awareness, research on hypertension among adolescents in Asia remains limited. While urban-rural disparities and general trends have been explored, less than 10% of studies address how gender shapes outcomes across diverse socioeconomic contexts [18]. This gap hinders public health efforts as unmanaged adolescent hypertension significantly increases the risk of cardiovascular disease (CVDs) in adulthood.

Early intervention can reduce the risk of heart disease and stroke by up to 30% [19]. Yet, the ongoing demographic and epidemiological shifts in developing Asian countries are giving rise to complex health challenges, particularly the increasing burden of non-communicable diseases among adolescents. As urbanisation and lifestyle transitions accelerate, the risk factors for hypertension are becoming more pervasive, yet comprehensive comparative research across diverse Asian contexts remains insufficient [20]. Through this systematic review, we aimed to address this critical gap by synthesising recent data from across Asia to offer an updated and regionally nuanced perspective of hypertension trends and risk factors among adolescents. By integrating findings from recent studies (2019–23) with rigorous methodological consistency, our findings could add substantial novelty, allowing for a clearer understanding of the regional disparities and emerging trends, and could thus inform the formulation of targeted and culturally appropriate public health interventions that can mitigate long-term cardiovascular risks for future generations.

## METHODS

### Search strategy

We conducted a comprehensive literature review to identify studies reporting the prevalence of elevated BP or hypertension among adolescents in Asia. We searched PubMed, Embase, ScienceDirect, Scopus, Web of Science, and Google Scholar for studies published between January 2019 and June 2024. The search terms comprised combinations of relevant keywords and variations in the names of 48 Asian countries, resulting in search strings like (“Hypertension” OR “Elevated blood pressure” OR “high blood pressure” OR “systolic hypertension” OR “diastolic hypertension”) AND (“Adolescents” OR “teenagers” OR “youth” OR “school”) paired with the names of each Asian country. We tailored the search strategies for each database to ensure the retrieval of relevant studies and reviewed the reference lists of eligible articles for additional sources. The search was restricted to articles published in the English language.

### Inclusion and exclusion criteria

To be included, a study had to report on the prevalence of hypertension or elevated BP, focus on adolescents aged 10–19 years, ensure that the target population aligns with the World Health Organization’s (WHO) definition of adolescence, be conducted within the geographical boundaries of Asia, have a cross-sectional design and report on both male and female participants, and be published between January 2019 and June 2024 (to ensure the use of recent data and trends).

We excluded studies focussed on populations outside Asia or age groups outside the adolescent age range of 10–19 years. We also omitted case reports, conference proceedings, review articles, policy briefs, and other secondary sources; studies without primary data or a clearly defined methodology; and studies focussing on specific clinical subgroups, such as adolescents with pre-existing chronic illnesses or those undergoing treatment for hypertension, in order to maintain focus on the general adolescent population.

We decided to include only cross-sectional studies to ensure methodological consistency and comparability of findings across the selected studies. Cross-sectional designs provide a snapshot of prevalence at a specific point in time, allowing for uniform comparisons between studies while minimizing the variability associated with different research methodologies.

### Data extraction

Two authors (BI and TII) screened titles, abstracts, and full texts against the inclusion criteria, resolving discrepancies through discussion or consultation with a third author (WT). We extracted data from eligible studies using a predefined extraction form, recording the author, publication year, and the country in which the research for each study. We further captured information on the sample size and participants' age range (10–19 years) to ensure that the studies were aligned with a focus on adolescents, as well as the gender distribution, *i.e.* the proportion of male and female participants. We classified the study setting as urban, rural, or mixed depending on the location where the research was conducted. We also retrieved any details regarding the number of BP readings, with many studies reporting averages from multiple measurements to ensure accuracy. We further documented the diagnostic criteria or guidelines used to define elevated BP or hypertension, such as the AAP 2017 guidelines or percentile-based BP thresholds. Finally, we recorded the prevalence of elevated BP or hypertension as reported in each study. Two reviewers independently extracted all data, resolving disagreements through discussion or consultation with a third reviewer to ensure consistency and accuracy in the extraction process.

### Quality assessment

We evaluated the methodological quality of included studies using a checklist developed by the Joanna Briggs Institute (JBI) for cross-sectional analytical studies [21]. This checklist evaluates studies based on several key criteria: the clarity of research questions, appropriate sampling methods, the reliability of outcome measures, risk of bias, and the quality of statistical analysis [22]. This process ensured a thorough and consistent evaluation of the studies included in this systematic review (Table S1 in the **Online Supplementary Document**). Our quality assessment indicated consistency in both the methodology and outcomes across the selected studies. However, some studies did have limitations in terms of reporting on potential confounding factors. The application of the JBI checklist showed that most studies were of high quality, but variations in methodology were noted [23–25].

## RESULTS

### Selection of studies

**Figure 1:**
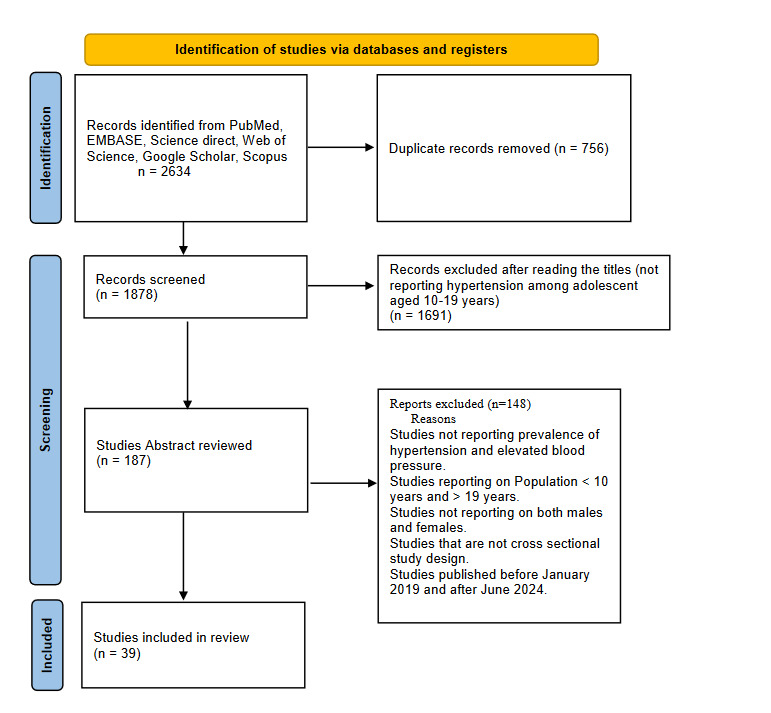
PRISMA flow diagram for the number of records identified from each database.

Our initial search retrieved 2634 records. After removing 756 duplicate entries, 1878 records remained for title and abstract screening. Following the exclusion of 1691 records that did not align with the eligibility criteria, we reviewed the full text of 187 records. Of these, 148 studies were excluded for various reasons, including a lack of primary data on the prevalence of hypertension, participants falling outside the targeted age range, reporting on only one gender, non-cross-sectional study designs, or being published outside the designated timeframe of January 2019 to June 2024. Thirty-nine studies met all the eligibility criteria and were included in this systematic review (**Figure 1**).

### Overview of included studies

**Figure 2:**
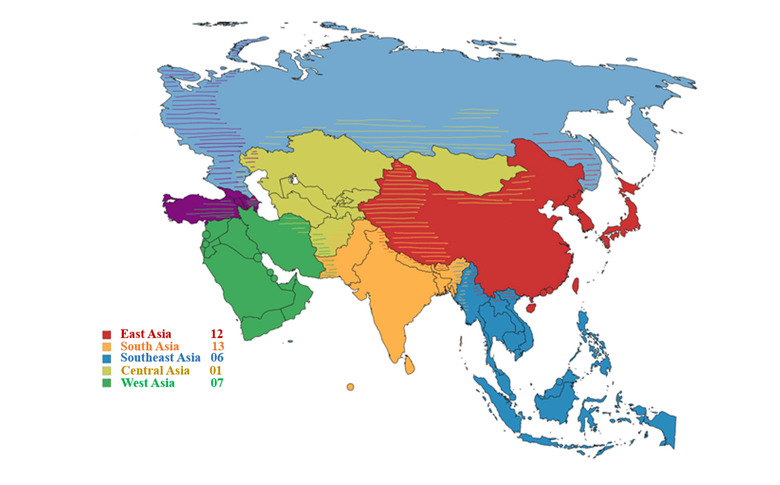
Geographical distribution of cross-sectional studies across various sub-regions in Asia.

Of the 39 studies from different sub regions across Asia (**Table 1**), 12 were conducted in East Asia (primarily in China and Korea), 13 in South Asia, six in Southeast Asia, seven in West Asia, and one in Central Asia (Kazakhstan), reflecting a wide range of cultural, economic, and environmental contexts (**Figure 2**). Their sample sizes varied significantly, ranging from 252 to 395 207 participants. Although some studies have focussed solely on one setting, a mix of urban and rural populations has also been reported. While most studies reported a relatively balanced sex distribution, a few displayed slight sex imbalances, typically with more male participants.

**Table 1:** Overview of selected articles

Author, year, country, reference	Sample size	Age range included in years	Male (%)	Female (%)	Urban/rural	Number of mean readings	Diagnostic criteria for hypertension	Prevalence of elevated BP/hypertension
**East Asia**								
Wang et al., 2022, China [26]	2916	10–18	67	33	Not specified	3	Hypertension defined as ≥130/80 mm Hg	Boys: 33.9%; girls: 32.0%
Park et al., 2024, Korea [27]	11 146	10–18	53.3	46.7	Urban: 70.3%, rural: 29.7%	3	2017 AAP guidelines (SBP≥130/80 mm Hg)	9.92% (overall for 2017–20 period) hypertension
Park et al., 2024, Korea [28]	2518	13–18	55.3	44.7	Mixed	3	EBP: >120/80; Hypertension: >130/80	Elevated BP: 5.1%; hypertensions: 3.1%
Liu et al., 2021, China [29]	8279	12v17	49.3	50.7	Mixed	3	High BP≥95th percentile	Normal high BP: 13.66%; high BP: 18.79%
Jiang et al., 2021, China [30]	12 849	17–19 (mostly 18.5)	44	56	Urban: 72.3%	3 (average of last two)	BP≥140/90 mm Hg	Hypertension: 4.3% (males: 7.9%; females: 1.6%)
Zhao et al., 2021, China [31]	1288	16.0–17.0	49.0	51.0	Not specified	3	BP≥95th percentile	Hypertension: 14.1% (males: 18.2%; females: 10.0%)
Zhou et al., 2022, China [32]	28 715	15–17	50.8	49.2	Urban/rural	3 readings averaged	2018 Chinese guidelines: 24.4%, 2017 AAP: 18.6%	Hypertension – males: 25.2% (CGC), 24.1% (AAP), 5.2% (CGA); females: 23.5% (CGC), 12.9% (AAP), 1.7% (CGA)
Liu et al., 2021, China [33]	42 025	12–17	49.6	50.4	Urban/rural	3 readings averaged	AAP 2017: 18.6% (initial), 5.9% (confirmed), CHL 2018: 24.5% (initial), 8.8% (confirmed)	AAP: 5.9%; CHL: 8.8%
Zou et al., 2019, China [34]	2639	12–15	53.8	46.2	Urban/rural	3 readings averaged	BP≥95th percentile	16.2% (male: 18.9%, female: 13.1%)
Heo et al., 2020, South Korea [35]	8755	10–17	52.9	47.1	Not specified	3 (average of 2nd and 3rd)	BP≥95th percentile	Elevated BP – boys: 8.3%, girls: 5.8%; hypertension – boys: 7.5%, girls: 6.1%
Sougawa et al., 2020, Japan [36]	1679	12–18	50.8	49.2	Urban	Not specified	Age- and sex-specific BP cut-offs by the Japanese Society of Hypertension	Females: 1.5%; Males: 3.1%
Zhang et al., 2019, China [37]	17 791	12–17	51.1	48.9	Urban/rural	2–3	National Blood Pressure Reference for Chinese Han Children (≥95th percentile)	Hypertension – male: 22.3%, female: 20.0%
**South Asia**								
Vasudevan et al., 2022, India [38]	11 718	10–19	51.5	48.5	Mixed	3	AAP guidelines	10–12 years: 35.1%; ≥13: 25.1% (high BP)
Kirti et al., 2023, India [39]	12 318	10–19	51.41	48.59	Not specified	Not specified	≥139/89 mm Hg for hypertension	Hypertension: 2.89%
Goswami et al., 2020, India [40]	1000 students	10–19	41.00%	58.90%	Urban	Not specified	Systolic BP ≥140 mmHg and/or diastolic BP ≥90 mmHg	Hypertension: 15.70%
Kala et al., 2021, India [41]	365 adolescents	10–19	61.4%	38.6%	Rural	Not specified	SBP and/or DBP ≥95th percentile	Hypertension: 17.5%
Akther et al., 2019, Bangladesh [42]	1000	12–16	NA	NA	Urban (Sylhet City)	Not specified	Hypertension defined as BP≥95th percentile	Hypertension: 0.70%
Islam et al., 2019, Bangladesh [43]	1146	10–17	NA	NA	Urban (Dhaka)	Not specified	Hypertension defined as BP≥95th percentile	Hypertension: 1.8% (male: 1.68%, female: 1.99%)
Mohan et al., 2019, India [44]	1959	11–17	50.5	49.5%	Urban/rural	2 (one week apart)	BP≥95th percentile	Urban: 8.4%, rural: 5.7%
Daniel et al., 2022, India [45]	864	10–19	56.1	43.9	Rural	3 (single visit)	AAP 2017 and NHBPEP guidelines	AAP 2017: 22.5% hypertension, NHBPEP: 15.2% hypertension
Mani et al., 2019, India [46]	932	13–17	71.6	28.4	Urban	1 (not averaged)	BP≥95th percentile	17.4% elevated BP
Banerjee et al., 2021 India, [47]	774	10–19	51.8	48.2	Urban slums	Average of 2 readings	APA 2017: BP≥95th percentile	Prehypertension: 10.6%; hypertension: 12.9%
Meitei et al., 2021, India (Northeast) [48]	728	15–19	64.5	35.5	Not specified	3 readings averaged	AAP 2017: ≥95th percentile, BP≥130/80 (Stage I & II)	Elevated BP: 20.47%; hypertension: 29.12%
Ravi et al., 2021, India [49]	252	11–16	68.7	31.3	Rural	2 (averaged)	BP≥95th percentile (height, gender-specific)	Pre-hypertension: 9.5%; HTN: 6%
Pereira et al., 2020, Pakistan [25]	288	13–16	42.4	57.6	Urban	Not specified	BP>95th percentile	Pre-hypertension: 9.4%; hypertension: 11.8%
**Southeast Asia**								
Tee et al., 2020, Malaysia [50]	513	12–16	41.1	58.9	Not specified	2	≥90th and ≥95th percentile thresholds based on age, sex, and height percentiles	Pre-hypertension: 19.1%; hypertension: 11.9% (stage 1 or 2)
Liew et al., 2019, Malaysia [51]	273	13–17	44	56	Urban (Johor)	3	BP≥95th percentile	Hypertension: 24.5%
Sudikno et al., 2023, Indonesia [52]	2735	15–19	48.2	51.8	Rural: 52.3%	At least 2 readings	BP≥95th percentile for adolescents; JNC VII for ages 18–19	Pre-hypertension: 16.8%; hypertension: 2.6%
Pirojsakul et al., 2022, Thailand [53]	3505	10–19	49.1	50.9	Urban/rural	3 (average of last 2)	2017 AAP guidelines: SBP or DBP≥95th percentile	Hypertension: 9.4%
Poh et al., 2022, Malaysia [54]	254	10–16	50	50	Urban (Kuala Lumpur)	2 (if needed, 3 readings)	NHBPEP guidelines. Hypertension: ≥95th percentile, prehypertension: 90th–95th percentile, or ≥120/80 mm Hg	Pre-hypertension: 3.9%; hypertension: 4.7%
Chai et al., 2021, Malaysia [55]	2461	13-17	42	58	Urban: 25.8%, rural: 74.2%	2 (or 3 if required)	4th Report on Diagnosis, Evaluation, and Treatment of High BP in Children and Adolescents	Prevalence of hypertension: 30.1%
**Central Asia**								
Kerimkulova et al., 2019, Kazakhstan [56]	1519	12–13	49.1	50.9	Urban schools	1	Hypertension: SBP or DBP>95th percentile; prehypertension: SBP or DBP between 90th–94th percentile	24.8% high normal BP; 12.4% hypertension
**West Asia**								
Amiri et al., 2019, Iran [57]	9715	15–19	53.9	46.1	Mixed	2	Prehypertension: SBP/DBP≥90th but <95th percentile; hypertension: SBP/DBP≥95th percentile	Pre-hypertension – urban boys: 29.24%, urban girls: 20.06%, rural boys: 32.05%, rural girls: 24.13%; hypertension – urban boys: 15.04%, urban girls: 9.06%, rural boys: 11.79%, rural girls: 11.60%
Batran et al. 2021, Palestine [23]	509	10v13	52.5	47.5	Urban schools	1 (Dynamap monitor)	≥95th percentile for SBP or DBP	Hypertension: 7.3%; pre-hypertension: 38.7%
Al-Farhan et al., 2020, Kuwait [24]	367	10.4 ± 0.4	47	53	Urban	2	SBP≥120 mm Hg or DBP≥80 mm Hg based on 2017 guidelines	23.3% elevated BP; SBP≥120 mm Hg: 21%; DBP ≥80 mm Hg: 21%
Çam et al., 2020, Turkey [58]	896	14–19	57.3	42.7	Urban: 34.6%	3	SBP or DBP≥95th percentile for sex, age, and height percentile	Pre-hypertension: 11.2%; hypertension: 14.8%
Al-Nuaim et al. 2022, Saudi Arabia [59]	380	15–19	52.4	47.6	Urban/rural	2 (mean used)	BP≥95th percentile	Males: 16.75%; females: 23.20%
Çakır et al., 2020, Turkey [60]	686	15–17	54.7	45.3	Urban	3 (average of readings)	BP≥95th percentile	Hypertension: 10.3%; pre-hypertension: 5.4%
Fırat et al., 2021, Turkey [61]	370	14–18	45.4	54.6	Not specified	3 readings averaged	BP>95th percentile (age-specific)	Pre-hypertension: 19.8%; hypertension: 14.8%

AAP – American Academy of Pediatrics, BP – blood pressure, DBP – diastolic blood pressure, CGC – Chinese Guidelines for Children, CHL – Chinese Hypertension Guidelines, EBP – elevated blood pressure, SBP – systolic blood pressure

Our quality assessment indicated consistency in both the methodology and outcomes across the selected studies. However, some studies did have limitations in terms of reporting on potential confounding factors. The application of the JBI checklist showed that most studies were of high quality, but variations in methodology were noted [23–25]. Following the criteria set forth by the Meta-Analysis of Statistics Assessment and Review Instrument (JBI-MAStARI), all included studies met the standards for high quality, confirming their suitability for inclusion in this review.

### Regional prevalence of hypertension among adolescents

**Figure 3:**
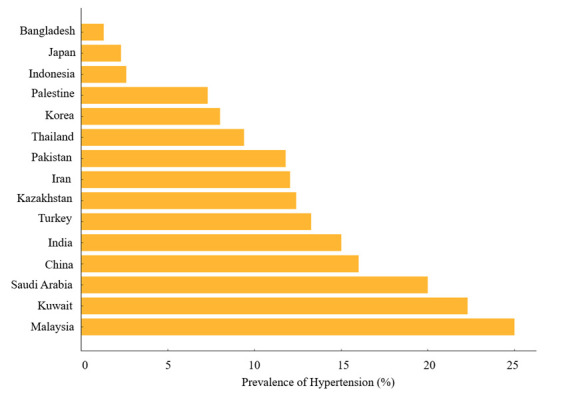
Country-specific prevalence of hypertension (2019–24).

The prevalence of adolescent hypertension varies notably across regions, with distinct differences based on geography and study settings. Prevalence rates ranged from 0.7% in Sylhet City, Bangladesh, to 24.5% in urban Malaysia. Diversity among the countries examined is reflected in the disparities observed within the sub-regions (**Figure 3**) [42,51].

**Figure 4:**
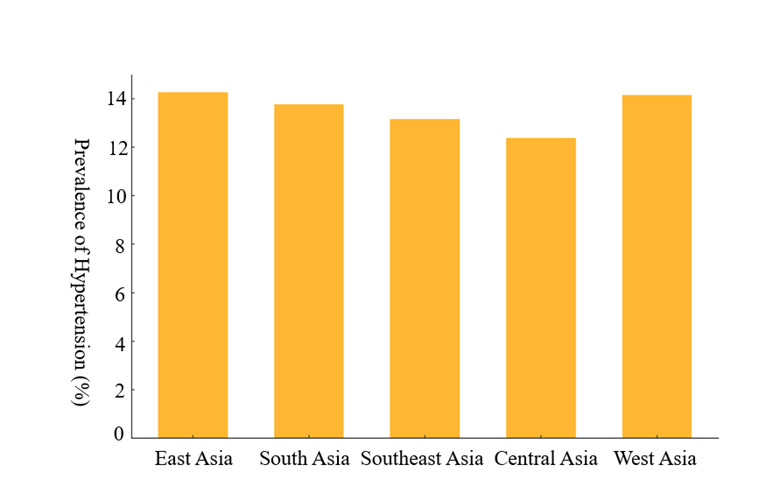
Prevalence of hypertension in different sub-region of Asia.

We observed substantial disparities in the prevalence of hypertension across regions, reflecting the influence of socioeconomic, dietary, and environmental factors (**Figure 4**). East Asia had the highest overall prevalence with an average rate of 14.25%, followed by West Asia (14.14%), South Asia (13.77%), Southeast Asia (13.16%), and Central Asia (12.37%).

**Figure 5:**
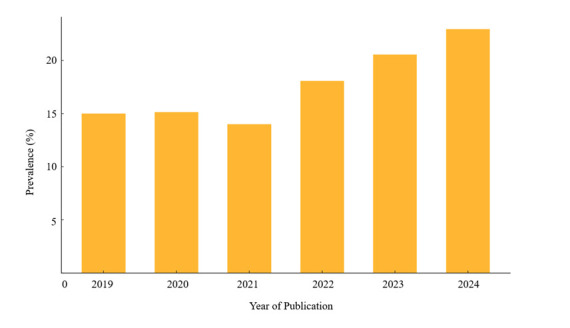
Prevalence of hypertension by year.

Our by-year analysis of the included studies shows a progressive increase in the overall prevalence of hypertension from 2019 to 2024 (**Figure 5**). Specifically, there was a clear gradual increase in prevalence in this period, with the rate rising from 15.0% in 2019 to 22.9% in 2024.

The prevalence rates varied significantly in East Asia. In China, a study of 28 715 adolescents aged 15–17 years reported hypertension rates of 24.4% using the 2018 Chinese guidelines and 18.6% based on the AAP 2017 criteria [32]. In Korea, the prevalence of hypertension among adolescents aged 10–18 years was 9.92% [27].

South Asia also had considerable variation in the prevalence of adolescent hypertension, with India demonstrating particularly high rates. A study conducted in India in 2022 among 11 718 adolescents aged 10–19 years reported a hypertension prevalence of 35.1% among adolescents aged 10–12 years and 25.1% among those aged 13 years and above [38]. These higher rates in India may be attributed to factors such as rapid urbanisation, socioeconomic disparities, and dietary shifts towards high-sodium processed foods, all of which contribute to elevated BP among young populations. In contrast, a study in Bangladesh reported a lower prevalence, with urban adolescents in Dhaka showing a prevalence of 1.8% [43].

Studies in Southeast Asia, specifically Malaysia, have explored the prevalence of hypertension in urban areas. One study encompassing 2461 Malaysian adolescents aged 13–17 years reported a 30.1% prevalence of hypertension [55]. Conversely, in Indonesia, the prevalence was comparatively low (2.6%) among adolescents aged 15–19 years old [52].

We further found gender and urban-rural disparities in the prevalence of hypertension in West Asia. In Iran, a study of 9715 adolescents aged 15–19 years reported hypertension prevalence rates of 15.04% in urban males and 9.06% in urban females [57]. Similarly, populations in Saudi Arabia had an elevated BP prevalence exceeding 35%, along with moderate hypertension rates, underscoring the urgent need for targeted interventions in this region [59].

In Central Asia, Kazakhstan has reported relatively high rates of elevated BP in adolescents. A study of 1519 adolescents aged 12–13 years found that 24.8% had elevated BP, serving as an early warning sign for future cardiovascular risks [56].

### Urban *vs.* rural disparities

**Figure 6:**
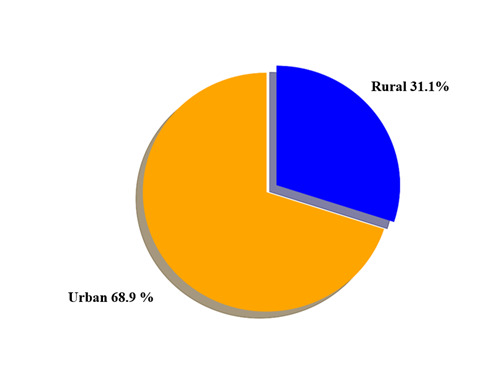
Weighted urban *vs.* rural focus in studies.

The prevalence of hypertension was generally higher in urban areas than in rural areas across multiple studies. For example, in India, a cross-sectional study of 1959 adolescents from urban and rural areas reported an 8.4% hypertension prevalence among urban adolescents *vs.* 5.7% among rural adolescents [44]. A similar trend was observed In Malaysia, where urban adolescents had a 24.5% prevalence of hypertension compared to lower rates in mixed or rural settings [51]. Most studies included in our review were from urban areas (**Figure 6**), so our findings highlight the potential influence of urban living, characterised by lifestyle factors, dietary habits, and reduced physical activity, on the prevalence of hypertension.

### Gender differences

We observed gender-based differences in hypertension prevalence, with several studies indicating higher rates among males. For example, in China, hypertension is more prevalent in males (22.3%) than in females (20%) [37]. Similarly, in Saudi Arabia, the prevalence of hypertension among urban male adolescents was 16.75%, compared to 11.79% among rural males [59]. These patterns align with international findings and may reflect behavioural and physiological differences, such as variations in body mass index (BMI) and physical activity levels.

### Diagnostic criteria and measurement protocols

**Figure 7:**
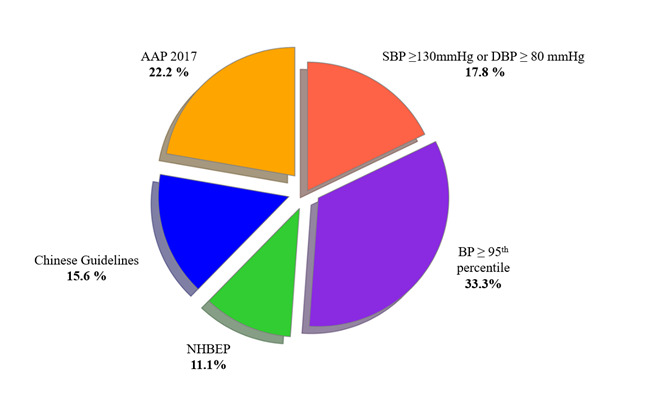
Proportion of studies using specific hypertension diagnostic guidelines.

The studies largely adhered to consistent BP measurement protocols, with most using three measurements averaged to ensure reliable results. Both mercury sphygmomanometers and digital devices were used, following either the national or AAP 2017 guidelines for diagnosing elevated BP or hypertension. We saw variability in the diagnostic criteria across studies, which influenced the reported prevalence rates. The following diagnostic criteria were applied most frequently (**Figure 7**):

- BP ≥95th percentile: used in 33.3% of the studies, this guideline often resulted in higher prevalence rates.
- AAP 2017 guidelines: adopted in 22.2% of the studies, defining hypertension at 130/80 mmHg for adolescents aged 13 years and older, contributing to more standardised comparisons.
- Chinese national guidelines: used in 15.6% of the studies, these guidelines resulted in prevalence rates ranging from 18.6% to 24.4% among Chinese adolescents.

The variability in diagnostic criteria significantly impacts the reported prevalence of hypertension among adolescents [62]. Studies using the ≥95th percentile threshold tend to report higher rates, which may distort regional comparisons [32]. These discrepancies can skew the understanding of hypertension burden and hinder effective public health interventions, as differing criteria might lead to either over- or underestimation of the condition [63]. Furthermore, such variations complicate cross-country comparisons, limiting the generalizability of findings and making it difficult to draw consistent public health recommendations [64]. To ensure more reliable and comparable data, standardising diagnostic criteria across regions is essential for accurate prevalence estimates and effective intervention planning.

## DISCUSSION

This systematic review is one of the first to synthesise recent data on elevated BP and hypertension among adolescents aged 10–19 years across Asia. Resulting from our analysis of data from 39 studies and a combined sample of over 200 000 adolescents from diverse Asian countries, our findings underscore that adolescent hypertension is a rising public health concern in this demographic, with some studies reporting hypertension prevalence rates as high as 24.5% in certain urban areas such as Malaysia. The higher prevalence of hypertension in urban adolescents can be attributed to factors such as increased consumption of fast food, lower physical activity, greater exposure to pollution, and higher stress levels from education-related pressures, all of which are more prevalent in urban settings [65]. These findings highlight the potential influence of lifestyle factors, dietary habits, and reduced physical activity on urban living [66].

The prevalence of adolescent hypertension in Asia shows substantial variation, with rates ranging from 2.6% in Indonesia [52] to 24.5% in Malaysia [67]. For instance, in East Asia, prevalence estimates varied from 5.9% in Korea (AAP≥130/80 mm Hg guideline) [27] to 24.4% in China [32]. These findings align with global prevalence estimates, where adolescent hypertension is reported to be approximately 11.2%, according to recent meta-analyses [3]. The observed differences between East and South Asian countries may be partially attributed to lifestyle and dietary factors. For example, urban areas in countries like India reported a hypertension prevalence as high as 22.5%, which is significantly above the global average and highlights the impact of lifestyle and environmental factors on adolescent BP [68].

The higher prevalence of hypertension in East Asia may be driven by dietary factors, such as increased sodium intake and the consumption of processed foods [69]. Genetic predispositions, coupled with more advanced health care infrastructure, may also contribute to better detection and reporting, distinguishing East Asia from Central and Southeast Asia where these factors are less pronounced [70].

Regional disparities in public health responses are also significant [71]. East Asia, particularly China and Korea, has well-established hypertension screening programmes [72,73], while Southeast Asia (*e.g.* Malaysia) faces implementation challenges, despite high prevalence [74]. South Asia, especially India, shows alarming rates but lacks uniform screening initiatives, and West Asia has begun addressing hypertension with regional collaborations, though rural areas remain underserved [75]. Comparisons with data from other regions, such as sub-Saharan Africa, provide additional context. Specifically, the prevalence of hypertension in sub-Saharan Africa was approximately 9.9% [76], which aligns with the lower end of the prevalence range observed in Asia. In West Asia, however, the prevalence among adolescents was higher, with Saudi Arabia reporting hypertension rates up to 23.2% in males, a trend comparable to findings in urban Malaysia [59]. Such regional differences likely reflect varying socioeconomic and lifestyle factors, including dietary patterns and physical activity levels. Importantly, the variability in diagnostic criteria, where some studies use absolute thresholds such as 130/80 mm Hg and others rely on percentile-based approaches, also contributes to differences in reported prevalence.

Socioeconomic status (SES) plays a critical role in hypertension prevalence, with lower SES often associated with higher rates in both urban and rural settings [77]. In urban areas, adolescents from higher SES backgrounds experience elevated rates of hypertension due to unhealthy lifestyles, while those from lower SES may have limited access to health care and early detection [78]. Rural populations, conversely, experience underdiagnosis due to barriers in health care access [79].

Sex-based differences in hypertension prevalence across Asian studies are consistent with international patterns, with male adolescents generally showing higher BP levels than females [80]. For example, studies in China reported hypertension rates of 22.3% in male adolescents compared to 20% in females, a trend similar to that observed in sub-Saharan Africa and other regions [81], such as the USA [82] and Europe [9,83,84]. The higher prevalence of hypertension in males may be attributed to several factors. Physiologically, elevated testosterone levels influence vascular tone and sodium retention, contributing to increased BP [85,86]. Behaviourally, males often exhibit higher salt consumption and lower health care-seeking behaviours, which can exacerbate hypertension [87]. Socially, gender differences in physical activity, with males tending to engage in less cardiovascular-friendly activities, may also contribute to the observed disparity [88]. North American studies support these findings, suggesting that adolescents with higher BMIs, particularly males, are at an increased risk of elevated BP [89].

The variability in BP measurements across studies, such as differences in devices (*e.g.* manual *vs.* digital) and number of readings (single *vs.* multiple), could introduce bias in reported prevalence rates [90]. For example, studies have shown that manual readings tend to result in higher BP levels compared to digital readings [90]. This can lead to overestimation of hypertension prevalences in those studies where manual readings were conducted. Previous studies that have directly compared these techniques have confirmed this; however, the use of randomization in these studies was rare [91]. Variations in timing (*e.g.* morning *vs.* afternoon) may also influence results, necessitating standardised protocols for more accurate and comparable estimates across regions.

Variations in diagnostic criteria used across studies represents a notable limitation in assessing prevalence. Approximately one-third of the studies in this review employed the ≥95th percentile criterion [25,27,29–31,34,35,37,43,44,46,50–52,56,57,59–61,92], while others used the AAP’s 130/80 mm Hg threshold for adolescents aged 13 and older [27,33,38,45,47,48,53,68]. Such differences contribute to the broad prevalence range, as studies utilising percentile-based thresholds often report slightly higher prevalence rates than those using fixed BP cut-off values. This discrepancy is consistent with findings from African [93–96] and Western studies [97,98], where absolute BP cutoffs tend to yield lower prevalence rates than percentile-based criteria [99]. Establishing uniform diagnostic guidelines would enhance comparability across regions and improve the accuracy of prevalence estimates in cross-regional studies [100]. The trends we observed here emphasise the urgent need for standardised screening and early intervention strategies across Asian countries. In high-prevalence areas such as urban centres in Malaysia and China, where rates exceed 20% [29,51], early detection and preventive interventions targeting high-risk groups could significantly mitigate long-term cardiovascular risks.

### Recommendations and strategies

The standardisation of diagnostic protocols in research and clinical settings is imperative to address the increasing prevalence of adolescent hypertension [101]. The current variation in diagnostic criteria, particularly between absolute and percentile-based thresholds, has led to inconsistencies that hinder the comparability of the prevalence data [102]. To minimise these discrepancies, we recommend adopting a unified approach using standards such as the AAP 2017 guidelines, which would improve the reliability of cross-regional assessments.

Targeted public health initiatives are also imperative, especially in high-prevalence urban areas where school-based BP screening programmes could facilitate early detection and intervention [103]. These initiatives should be supplemented by public health campaigns that focus on lifestyle education, promoting balanced diets, physical activity, and stress management among adolescents and their families. Additionally, Policies should be enacted to regulate salt and sugar intake in schools and public spaces, while also mandating physical activity education to encourage healthier habits.

Public health campaigns focussing on lifestyle education, such as promoting balanced diets, physical activity, and stress management, are recommended to foster preventive behaviours among adolescents and their families [104]. Efforts should particularly target low- and middle-income regions, where tailored interventions are most needed. Specific strategies must be developed to address the unique needs of high-risk groups, such as males and adolescents in urban settings. Collaboration between governments, health care providers, and educational institutions will be essential for the successful implementation of these targeted interventions across Asia.

### Limitations

While this review provides valuable insights, it is limited by the variability in BP measurement protocols and diagnostic criteria, which limits comparability and generalisability of the findings. Also, our reliance on cross-sectional studies limits causal interpretations. We opted to focus solely on cross-sectional studies to ensure homogeneity in study design and to allow for more consistent comparisons across studies. However, this restricted our ability to examine the development and progression of hypertension in adolescents over time. While longitudinal studies would provide valuable insights, they are relatively scarce in this area of research. Additionally, the geographic concentration of studies in East and South Asia limits the generalisability across all Asian sub-regions. Many studies have focussed predominantly on urban populations, which may not reflect rural health patterns, particularly when economic and lifestyle factors differ substantially. Potential publication bias should be considered, as studies with lower prevalence rates may be underreported, which could lead to an underestimation of hypertension prevalence in adolescents across Asia. Future studies should aim for longitudinal studies with broader regionally representative samples and implement standardized protocols to improve comparability.

## CONCLUSIONS

This systematic review underscores the significant and rising prevalence of hypertension among adolescents in Asia, which ranges from 0.7% in urban Bangladesh to 24.5% in urban Malaysia. Male adolescents consistently showed higher hypertension rates, reaching 22.3% in China compared to 20% in females. There was also a noticeable urban-rural disparity, with urban adolescents showing rates up to 8% higher than their rural counterparts. The findings also highlight the critical role of socioeconomic factors, where urban populations, particularly in wealthier regions, experience higher hypertension rates due to lifestyle factors such as poor diet and limited physical activity. Tailored interventions that address socioeconomic disparities and ensure equitable access to health care across regions will be crucial to combating adolescent hypertension effectively.

## Additional material


Online Supplementary Document
