# Supplementary material for: Current status of elevated blood pressure and hypertension among adolescents in Asia: a systematic review
Source: J Glob Health. 2025 Mar 28;15:04115. doi: 10.7189/jogh.15.04115 (PMC11949511; doi:10.7189/jogh.15.04115)
Supplement: Online Supplementary Document [file jogh-15-04115-s001.pdf]

Supplement to: Islam B, Tasiu II, Wang T, Wu M, Qin J. Current status of elevated blood pressure and hypertension among adolescents in Asia: a systematic review. J Glob Health. 2025;15:04115.

Table S1: Assessment of Bias Risk in the Included Studies Using JBI-MAStARI

Author, Year, Ref	Q1	Q2	Q3	Q4	Q5	Q6	Q7	Q8	Yes Score (_/8) Methodological quality
Jinling Wang et al. 2022 [19]	Y	Y	Y	Y	Y	Y	Y	Y	8/8
Park et al. 2024 [20]	Y	Y	Y	Y	Y	Y	N	Y	7/8
Park et al. 2024 [21]	Y	Y	Y	Y	Y	Y	N	Y	7/8
Liu et al. 2021 [22]	Y	Y	Y	Y	Y	Y	N	Y	7/8
Qingqing Jiang et al. 2021 [23]	Y	Y	Y	Y	Y	Y	Y	Y	8/8
Weiying Zhao et al. 2021 [24]	Y	Y	Y	Y	Y	Y	N	Y	7/8
Haoqi Zhou et al. 2022 [25]	Y	Y	Y	Y	Y	Y	Y	Y	8/8
Kai Liu et al. 2021 [26]	Y	Y	Y	Y	Y	Y	N	Y	7/8
Yunfei Zou et al. 2019 [27]	Y	Y	Y	Y	Y	Y	Y	Y	8/8
Somi Heo et al. 2020 [28]	Y	Y	Y	Y	Y	Y	Y	Y	8/8
Yukiko Sougawa et al. 2020 [29]	Y	Y	Y	Y	Y	Y	Y	Y	8/8
Xiyan Zhang et al. 2019 [30]	Y	Y	Y	Y	Y	Y	Y	Y	8/8
Vasudevan et al. 2022 [31]	Y	Y	Y	Y	Y	Y	Y	Y	8/8
Kirti et al. 2023 [32]	Y	Y	Y	Y	Y	Y	Y	Y	8/8
Goswami et al., 2020, India [33]	Y	Y	Y	Y	Y	Y	Y	Y	8/8
Kala et al., 2021, India [34]	Y	Y	Y	Y	Y	Y	Y	Y	8/8
Akther et al. 2019 [35]	Y	Y	Y	Y	Y	Y	Y	Y	7/8
M R Islam et al. 2019 [36]	Y	Y	Y	Y	Y	Y	Y	Y	7/8
Bishav Mohan et al. 2019 [37]	Y	Y	Y	Y	Y	Y	Y	Y	8/8
Roy Arokiam Daniel et al. 2022 [38]	Y	Y	Y	Y	Y	Y	N	Y	7/8
Geetha Mani et al. 2019 [39]	Y	Y	Y	Y	Y	Y	Y	Y	8/8
Sitikantha Banerjee et al. 2021 [40]	Y	Y	Y	Y	Y	Y	Y	Y	8/8
Sanjenbam Yaiphaba Meitei et al. 2021 [41]	Y	Y	Y	Y	Y	Y	Y	Y	8/8
Ramya Kundayi Ravi et al. 2021 [42]	Y	Y	Y	Y	Y	Y	Y	Y	8/8
Felicianus A. Pereira et al. 2020 [43]	Y	Y	Y	Y	Y	Y	Y	Y	8/8

Author, Year, Ref	Q 1	Q2	Q3	Q4	Q5	Q6	Q7	Q8	Yes Score (_/8) Methodological quality
Joyce Ying Hui Tee et al. 2020 [44]	Y	Y	Y	Y	Y	Y	Y	Y	8/8
Liew JK et al. 2019 [45]	Y	Y	Y	Y	Y	Y	Y	Y	8/8
Sudikno et al. 2023 [46]	Y	Y	Y	Y	Y	Y	Y	Y	8/8
	Y	Y	Y	Y	Y	Y	Y	Y	8/8
Kwanchai Pirojsakul et al. 2022 [47]	Y	Y	Y	Y	Y	Y	Y	Y	8/8
Poh et al. 2022 [48]	Y	Y	Y	Y	Y	Y	Y	Y	8/8
Chaiet al. 2021 [49]	Y	Y	Y	Y	Y	Y	N	Y	7/8
Kerimkulova et al. 2019 [50]	Y	Y	Y	Y	Y	Y	Y	Y	8/8
Parisa Amiri et al. 2019 [51] 2019	Y	Y	Y	Y	Y	Y	Y	Y	8/8
Batran et al. 2021 [52]	Y	Y	Y	Y	Y	Y	Y	Y	8/8
Al-Farhan et al. 2020 [53]	Y	Y	Y	Y	Y	Y	Y	Y	8/8
Hasan Hüseyin Çam et al. 2020 [54]	Y	Y	Y	Y	Y	Y	Y	Y	8/8
Anwar Al-Nuaim et al. 2022 [55]	Y	Y	Y	Y	Y	Y	Y	Y	8/8
Orhan Çakır et al.2020 [56]	Y	Y	Y	Y	Y	Y	N	Y	7/8
Fırat et al.[57] 2021	Y	Y	Y	Y	Y	Y	N	Y	7/8

The items were collapsed into 8 quality-appraisal criteria (Q1-Were the criteria for inclusion in the sample clearly defined? Q2-Were the study subjects and the setting described in detail? Q3-Was the exposure measured in a valid and reliable way? Q4-Were objective standard criteria used for measurement of the condition? Q5Were confounding factors identified? Q6-Were strategies to deal with confounding factors stated? Q7-Were the outcomes measured in a valid and reliable way? Q8Was appropriate statistical analysis used?).

JBI-MAStARI was used to evaluate the risk of bias. Articles with scores between 1 and 2 were defined as poor methodological quality, articles with scores between 3 and 4 as moderate quality, and articles with scores more than 5 as high quality.
N, no; NA = not relevant; U = unsure; Y = yes.
